# Comparative study of the effect of solvents on the efficacy of neonicotinoid insecticides against malaria vector populations across Africa

**DOI:** 10.1186/s40249-022-00962-4

**Published:** 2022-04-25

**Authors:** Magellan Tchouakui, Tatiane Assatse, Leon M. J. Mugenzi, Benjamin D. Menze, Daniel Nguiffo-Nguete, Williams Tchapga, Jonathan Kayondo, Francis Watsenga, Emile Zola Manzambi, Michael Osae, Charles S. Wondji

**Affiliations:** 1 Centre for Research in Infectious Diseases (CRID), P.O. Box 13501, Yaoundé, Cameroon; 2 Parasitology and Ecology Laboratory, Department of Animal Biology and Physiology, Faculty of Science, University of Yaoundé 1, P.O. Box 812, Yaoundé, Cameroon; 3 Institut National de Recherche Biomédicale, Kinshasa, Democratic Republic of Congo; 4 Radiation Entomology and Pest Management Centre, Biotechnology and Nuclear Agriculture Research Institute, Ghana Atomic Energy Commission, P.O. Box LG80, Legon-Accra, Ghana; 5 Department of Vector Biology, Liverpool School of Tropical Medicine, Pembroke Place, Liverpool, L35QA UK; 6 International Institute of Tropical Agriculture (IITA), P.O. Box 2008, Yaoundé, Cameroon; 7 Entomology Department, Uganda Virus Research Institute (UVRI), P.O. Box 49, Entebbe, Uganda

**Keywords:** Malaria, Anopheles, Insecticide resistance, Neonicotinoids, Clothianidin, Cross-resistance

## Abstract

**Background:**

New insecticides with a novel mode of action such as neonicotinoids have recently been recommended for public health by WHO. Resistance monitoring of such novel insecticides requires a robust protocol to monitor the development of resistance in natural populations. In this study, we comparatively used three different solvents to assess the susceptibility of malaria vectors to neonicotinoids across Africa.

**Methods:**

Mosquitoes were collected from May to July 2021 from three agricultural settings in Cameroon (Njombe-Penja, Nkolondom, and Mangoum), the Democratic Republic of Congo (Ndjili-Brasserie), Ghana (Obuasi), and Uganda (Mayuge). Using the CDC bottle test, we compared the effect of three different solvents (ethanol, acetone, MERO) on the efficacy of neonicotinoids against *Anopheles gambiae* s.l. In addition, TaqMan assays were used to genotype key pyrethroid-resistant markers in *An. gambiae* and odds ratio based on Fisher exact test were used to evaluate potential cross-resistance between pyrethroids and clothianidin.

**Results:**

Lower mortality was observed when using absolute ethanol or acetone alone as solvent for clothianidin (11.4‒51.9% mortality in Nkolondom, 31.7‒48.2% in Mangoum, 34.6‒56.1% in Mayuge, 39.4‒45.6% in Obuasi, 83.7‒89.3% in Congo and 71.1‒95.9% in Njombe pendja) compared to acetone + MERO for which 100% mortality were observed for all the populations. Similar observations were done for imidacloprid and acetamiprid. Synergist assays (PBO, DEM and DEF) with clothianidin revealed a significant increase of mortality suggesting that metabolic resistance mechanisms are contributing to the reduced susceptibility. A negative association was observed between the L1014F-*kdr* mutation and clothianidin resistance with a greater frequency of homozygote resistant mosquitoes among the dead than among survivors (*OR* = 0.5; *P* = 0.02). However, the I114T-GSTe2 was in contrast significantly associated with a greater ability to survive clothianidin with a higher frequency of homozygote resistant among survivors than other genotypes (*OR* = 2.10; *P* = 0.013).

**Conclusions:**

This study revealed a contrasted susceptibility pattern depending on the solvents with ethanol/acetone resulting to lower mortality, thus possibly overestimating resistance, whereas the MERO consistently showed a greater efficacy of neonicotinoids but it could prevent to detect early resistance development. Therefore, we recommend monitoring the susceptibility using both acetone alone and acetone + MERO (4 µg/ml for clothianidin) to capture the accurate resistance profile of the mosquito populations.

**Graphical Abstract:**

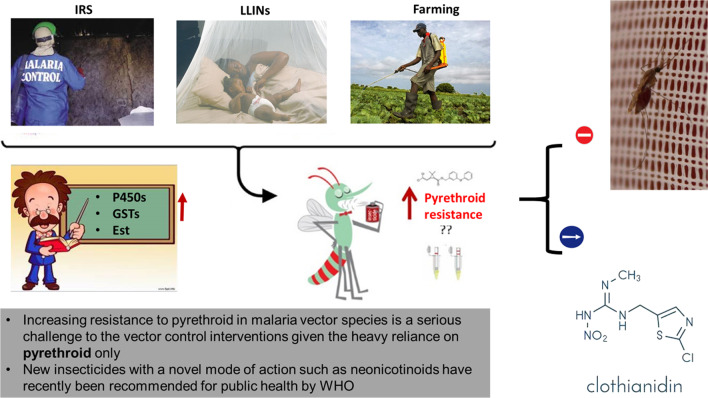

**Supplementary Information:**

The online version contains supplementary material available at 10.1186/s40249-022-00962-4.

## Background

Malaria prevention relies extensively on mosquito control using pyrethroid-based interventions including indoor residual spraying (IRS) and long-lasting insecticidal nets (LLINs) [1, 2]. The scale-up of these tools has significantly contributed to the important reduction of malaria burden in the past decade [1, 3]. However, increasing resistance to pyrethroid in malaria vector species is a serious challenge to these vector control interventions given the heavy reliance on pyrethroid only [4]. Most African *Anopheles gambiae* and *An. funestus* populations are resistant to pyrethroid insecticides and also show varying levels of resistance to other insecticides used for vector control (carbamates, organophosphates and organochlorines). Due to the threat posed by insecticide resistance, there has been an urgent call for alternative insecticides to supplement malaria vector control [5]. Novel insecticides are gradually being introduced by manufacturers and recommended by World Health Organization (WHO) for vector control [6]. Among these, the neonicotinoids (e.g. clothianidin) and pyrole (e.g. chlorfenapyr) are the new mode of action insecticide classes for public health, recently recommended by WHO for LLINs and IRS [7].

Clothianidin is a neonicotinoid insecticide that is chemically similar to nicotine. It acts on the central nervous system of insects as an agonist of acetylcholine and stimulates nicotine acetylcholine receptors (nAChR) [8] activating post-synaptic acetylcholine receptors but does not inhibit acetyl cholinesterase (ACh). High levels overstimulate and block the receptors [9], causing paralysis and death [8]. Clothianidin is the active ingredient in SumiShield (developed by Sumitomo Chemical Company, Japan) and Fludora® Fusion (Bayer CropScience, Monheim, Germany) along with deltamethrin, an IRS formulation which was recently added to the WHO prequalification list of recommended insecticides (https://www.who.int/pq-vector-control/ prequalifed-lists/en/).

As new insecticides are developed, it is essential to monitor the development of resistance to prolong their efficacy as much as possible and avoid the level of widespread resistance now seen with pyrethroids [3]. Such resistance monitoring requires establishing diagnostic concentrations to determine baseline susceptibility of malaria vectors and to enable surveillance of insecticide resistance once the insecticides are in use.

Nowadays, WHO protocol to test the susceptibility to neonicotinoids in mosquitoes is not well established thus, making resistance monitoring to this insecticide class very challenging. Clothianidin (neonicotinoid; IRAC MoA class 4A) for example, tends to crystallize if used in straight acetone/ethanol (solvents commonly used in bioassays) and the uptake of active ingredient between the insect’s body and the crystal is very low [10–12]. This has made the design of standard protocols more arduous compared to other insecticide classes. In addition, because this active ingredient acts slowly, resistance profiles can be detected after bioassays only if exposed populations are rigorously monitored during a long holding period, which can last for seven days or more. Despite these difficulties, the susceptibility of wild *Anopheles* populations from several African countries has been evaluated using 150 µg/ml of clothianidin dissolved in either absolute ethanol or acetone [10–13]. Recently, Bayer CropScience Ltd. introduced an 81% Rapeseed oil methyl ester (0.11% MERO®) which, when added to acetone, prevents the crystallization then keeps the clothianidin for a longer period in a solved state and allows therefore the feasibility of the bottle assay. A study conducted in ivory coast, established 50 µg/ml as diagnostic dose of clothianidin [14]. However, this study did not evaluate the lower doses to see if 50 µg/ml cannot mask the detection of resistance highlighting the need of stabilising the protocol for testing of neonicotinoids. In this study, we used three different solvents to comparatively evaluate the efficacy of neonicotinoids on malaria vectors from many African countries and established the diagnostic dose of clothiandin using acetone and MERO. Furthermore, we evaluated a potential cross-resistance between pyrethroids and clothianidin.

## Methods

### Study sites

Mosquitoes were collected from 4 regions across the continent. Mosquitoes were collected in three agricultural settings in Cameroon (Mangoum, Nkolondom, and Njombe-Penja) from May to July 2021. The climate is made up of two wet and two dry seasons typical of tropical climate around the equator. In the Democratic Republic of Congo (DRC) mosquitoes were collected at Ndjili Brasserie, a suburb of Kinshasa (4°19′39″S, 15°18′48″E), in June 2021. In Uganda, mosquitoes were collected in the Eastern region (May 2021), Mayuge (0°23′10.8′′N, 33°37′16.5′′E), and in Ghana collections were done in Obuasi (5°56′N, 1°37′W) in July 2021. In Cameroon, immature stages were collected from the breeding site using the dipping method whereas in other countries indoor resting blood fed females were collected using electric aspirators. Emerging adults (2‒5 days old) from collected larvae or F_1_ progeny (2‒5 days old) from indoor collected females were used for the bioassays.

### Molecular identification

The genomic DNA was extracted from a subset of mosquitoes from each of the collection sites using the Livak method [25], then the members of the *An. gambiae* complex were identified by the PCR [26, 27].

### Determination of susceptibility to neonicotinoids and establishment of the diagnostic dose of clothianidin using acetone + MERO as solvent

Clothianidin, imidacloprid and acetamiprid used were technical materials from Sigma (PESTANAL®, analytical standard, Sigma-Aldrich, Dorset, United Kingdom). These chemicals were mixed in three different solvents (acetone, absolute ethanol, or acetone with MERO). For acetone and ethanol alone, the dose of 150 µg/ml for clothianidin, 200 µg/ml for imidacloprid and 75 µg/ml for acetamiprid were used as previously described [11].

For acetone dissolved in MERO, a stock solution of an acetone/MERO® mixture was made by pipetting 0.11 ml (110 µl) MERO® to 100 ml of acetone (0.11%). 900 µg straight clothianidin was weighed and mixed with 10 ml of this stock solution of MERO plus acetone. After complete dissolution of the active ingredient, 10 ml acetone/MERO® solution containing 900 µg clothianidin was prepared. One [1] ml of this solution was applied to each test bottle to achieve a concentration of 90 µg clothianidin/250 ml bottle. This dose of insecticide was used to characterise the susceptibility of different mosquito populations. In addition, ranges of insecticide concentrations were tested (0.25, 0.50, 1, 2, 4, 40 and 90 µg/ml) using the susceptible lab strain Kisumu to evaluate the diagnostic dose of clothianidin when diluted in acetone and MERO. Approximately 24 h after coating bottles with insecticide, 25 female Kisumu (3–5 days old) were exposed to the insecticides for 1 h and the knocked down mosquitoes were recorded at the end of the 60 min (Kd-60) exposure period. After recording the Kd-60 mosquitoes were gently aspirated from the bottle into clean paper cups and provided with 10% sugar solution soaked in cotton wool during the recovery period and the final mortality was recorded 24 h post-exposure.

### Synergist assay with piperonyl butoxide (PBO), di-ethyl Maleate (DEM) and s,s,s–tri-butylphosphorotrithioate (DEF)

To identify the possible enzyme systems involved in reduced susceptibility to neonicotinoids, synergist bioassays were conducted for clothianidin in Nkolondom using the emerging adult from larval collection. Two- to four-day-old F_0_/F_1_ females were first exposed to the synergist (4% PBO, 8% DEM or 0.25% DEF) for 1 h, followed by exposure to 150 µg/ml clothianidin (dissolved in acetone) for 1 h. Mortality was recorded 24 h after exposure and the differences in mortalities between synergized and non-synergized experiments were compared using a Chi-square test.

### Potential cross-resistance between neonicotinoids and pyrethroids

To assess the potential cross-resistance between neonicotinoids and pyrethroids, we crossed the pyrethroid highly resistant field strain from Nkolondom (where the 1014F-*Kdr* is fixed) with the fully susceptible laboratory strain Kisumu (with no 1014F-*Kdr*). This hybrid strain was exposed to sub-lethal doses of clothianidin (dissolved in acetone only) to select the dead and alive mosquitoes. These mosquitoes were genotyped for the L1014F target-site knockdown resistance (*Kdrw*) and the 114 T-GSTe2 metabolic resistance marker (all associated with DDT/pyrethroid resistance in *An. gambiae*) using Taqman methods as previously described [15, 16]. PCR reactions (10 μl) contained 1 μl of genomic DNA, 5 μl of SensiMix DNA kit (catalog: SM2-717104), 0.125 μl of each probe and 3.875 μl of sigma water. Samples were run on a Mx3000P™ Multiplex quantitative PCR system with the temperature cycling conditions of: 10 min at 95 °C followed by 40 cycles of 95 °C for 10 s and 60 °C for 45 s.

Odds ratio and Fisher exact test were used to establish the statistical significance of any association between this DDT/pyrethroid resistance marker and the ability to survive clothianidin exposure.

### Data analysis

GraphPad Prism 7.00 was used for a construction of graphs. Chi-square test was used to compare the mortality rate between different treatments whereas odds ratio and Fisher exact test were used to establish statistical significance of any association between pyrethroid resistant markers and mosquitoe's ability to survive clothianidin exposure.

## Results

### Molecular identification of mosquitoes tested

PCR assays revealed that all the mosquitoes tested from Mangoum (44/44), Nkolondom (50/50), and Congo (60/60) were *An. gambiae*. Those collected in Njombe (Cameroon) were mainly *An. coluzzii* (58/60). The *An. gambiae* s.l. population from Uganda were mix of 82% *An. gambiae* and 17% *An. arabiensis* whereas those from Ghana were 60% (39/65) *An. gambiae* and 40% (39/65) *An. coluzzii.*

### Susceptibility profile to clothianidin

**Fig. 1:**
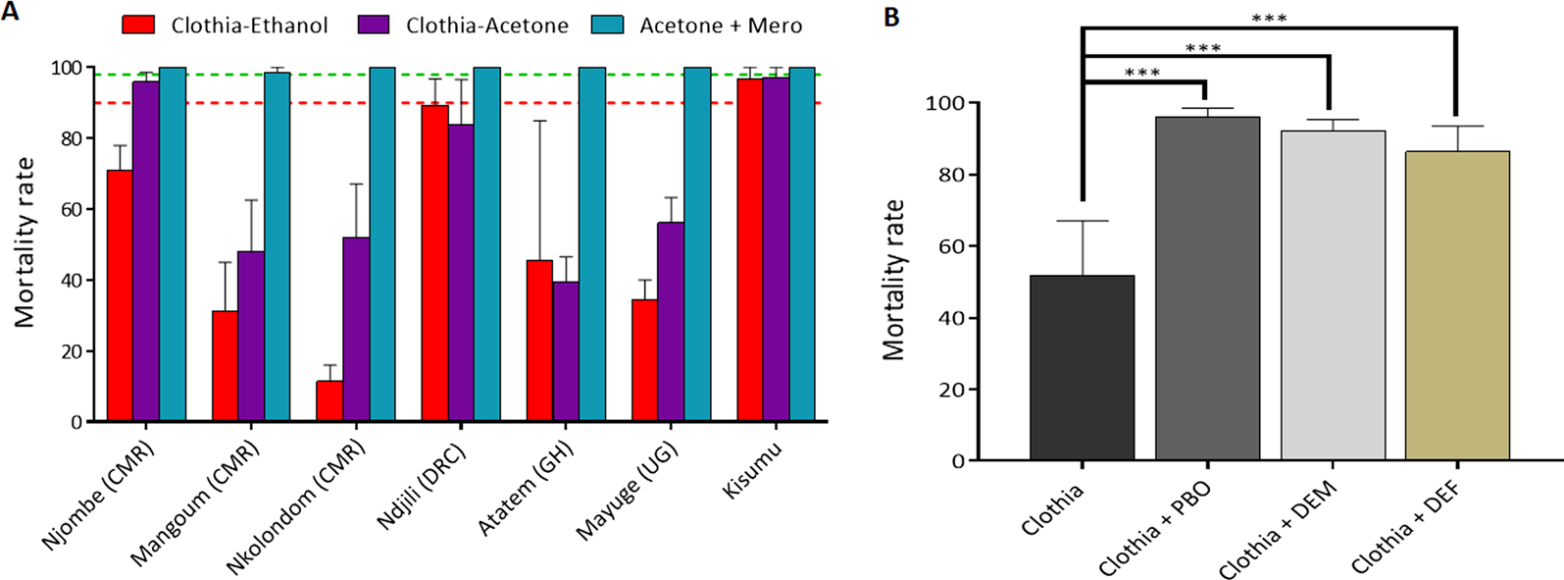
Susceptibility profile of *An. gambiae* s.l. to clothianidin across Africa. **A** Mortality rate (%) of mosquitoes from various sites 7 days post-exposure to clothianidin dissolved in different solvents compared to the susceptible lab strain Kisumu. **B** Effect of pre-exposure to synergist PBO, DEM and DEF against clothianidin on *An. gambiae* from Nkolondom. Results are average of percentage mortalities from four–five replicates each. The bars represent the standard error on the mean (SEM), linear color dots indicate the threshold for resistance (red) and susceptibility (green). *CMR* Cameroon, *DRC* Democratic Republic of Congo, *GH* Ghana, *UG* Uganda

The Kisumu lab strain was susceptible to clothianidin whatever the solvent used (Fig. 1A and Additional file 1: Fig. S1). However, the susceptibility to this insecticide varied significantly in *An. gambiae* field populations depending on the solvent used. Using acetone combined with 0.11% MERO® (81% Rapeseed oil methyl ester) as solvent, induced significant higher mortality compared to acetone or ethanol alone (Fig. 1A). A full susceptibility was observed for all the *An. gambiae* populations with a mortality rate of 100% when exposed to clothianidin dissolved in acetone + MERO at a concentration of 90 µg/ml (Fig. 1A). However, when exposed to clothianidin dissolved in acetone alone (150 µg/ml), the mortality varied from 51.1 ± 15.2% in Nkolondom to 95.9 ± 2.6% in Njombe-Penja. On the other hand, when using ethanol as solvent, the mortality varied from 11.4 ± 4.6% in Nkolondom to 71.0 ± 6.9% and 89.3 ± 7.6% in Njombe-penja and Ndjilli (DRC) respectively (Fig. 1A). The mortality with these two solvents increased significantly from 24 h to 7 days post-exposure confirming a slow-acting effect of this insecticide (Additional file 1: Fig. S1, Additional file 2: Fig. S2 and Additional file 3: Fig. S3).

### Susceptibility profile to imidacloprid

**Fig. 2:**
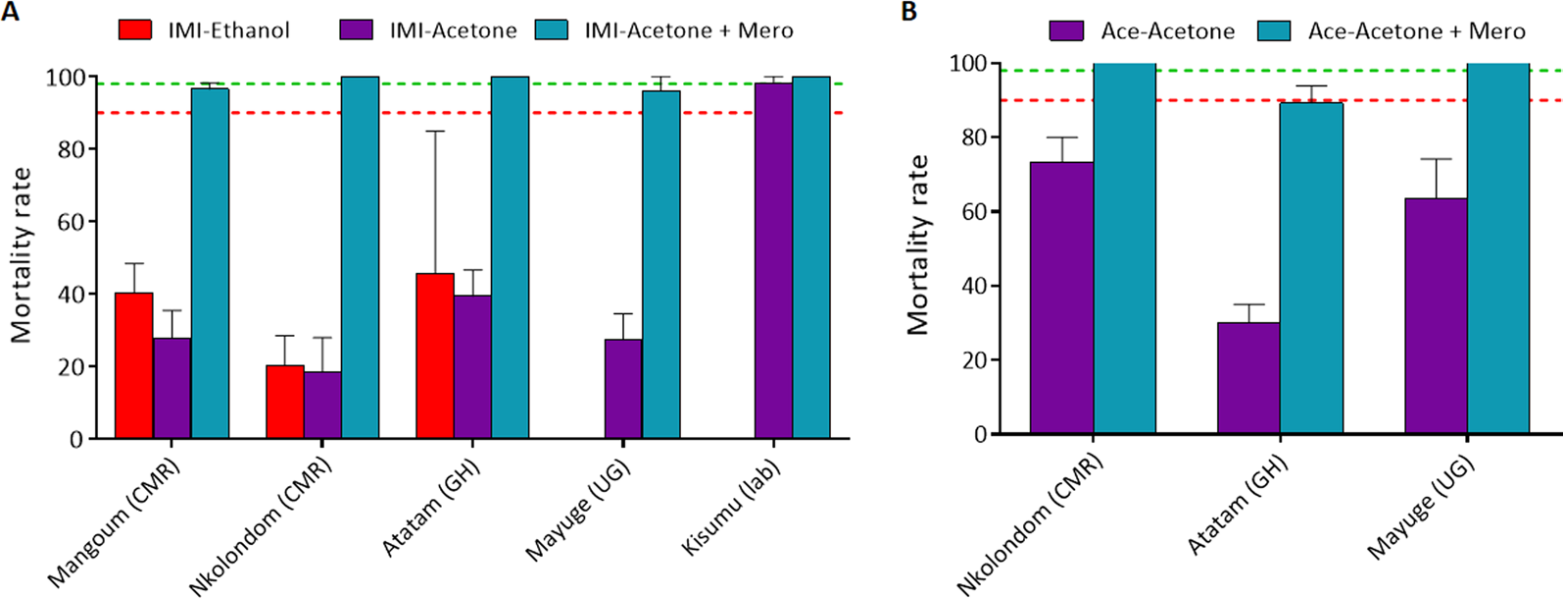
Susceptibility profile of *An. gambiae* s.l. to imidacloprid and acetamiprid across Africa. Mortality rate (%) of mosquitoes from different sites 7 days post-exposure to imidacloprid **A** and acetamiprid **B** dissolved in various solvents compared to the susceptible lab strain Kisumu. Results are average of percentage mortalities from four–five replicates each ± SEM. Linear colour dots indicate the threshold for resistance (red) and susceptibility (green). CMR: Cameroon; GH: Ghana; UG: Uganda

The susceptibility to imidacloprid was evaluated on mosquitoes from Nkolondom and Mangoum (Cameroon), Obuasi (Ghana) and Mayµge (Uganda). Higher mortality with this insecticide was observed in all the localities when using acetone + MERO as solvent. In Nkolondom, a mortality rate of 83.9% was observed with acetone + MERO compared to 65.3 ± 16.6% with acetone only (*χ*^2^ = 10.9; *P* = 0.001) and 27.2% with ethanol (*χ*^2^ = 64.8; *P* < 0.0001) suggesting a likely resistance in this population (Fig. 2A). The similar pattern was observed in Mangoum where a mortality rate of 96.6% was observed with acetone + MERO compared to 27.7% with acetone only (*χ*^2^ = 100; *P* < 0.0001) and 40.4% with absolute ethanol (*χ*^2^ = 72.8; *P* < 0.0001) (Fig. 2A). In Ghana, imadacloprid dissolved in acetone + MERO induced 97.9% mortality and 57.2% when dissolved in acetone only (*χ*^2^ = 47.3; *P* < 0.0001). The mortality was very low when using absolute ethanol as solvent with mortality rate of 12.6% (*χ*^2^ = 146; *P* < 0.0001). The similar profile (96.1% mortality vs. 27.3%) was obtained in Uganda (*χ*^2^ = 99.6; *P* < 0.0001).

### Susceptibility profile to acetamiprid

When dissolved in acetone + MERO, acetamiprid also displayed greater efficacy compared to when dissolved in acetone only (Fig. 2B). In Nkolondom, 100% mortality was obtained when using acetone and MERO compared to 73.5 ± 6.6% without MERO (*χ*^2^ = 30.4; *P* < 0.0001). In Obuasi, 89.0 ± 4.6% mortality was obtained when using acetone and MERO compared to 30% without MERO (*χ*^2^ = 71.8; *P* < 0.0001) (Fig. 2B). In Mayuge, full susceptibility was noticed with MERO compared to 60% mortality without MERO (*χ*^2^ = 49.7; *P* < 0.0001). These results suggest that the level of reduced susceptibility to acetamiprid is higher in Ghana compared to other locations tested.

### Synergist test

Susceptibility testing using PBO, DEM, and DEF as synergists revealed a significant recovery of susceptibility to clothianidin in Nkolondom which showed a highest level of reduced susceptibility to this insecticide when diluted in ethanol or acetone alone (Fig. 1B). In Nkolondom, the mortality with clothianidin + PBO was 96.0 ± 2.6% versus 51.9 ± 8.5% for clothianidin without PBO pre-exposure (*χ*^2^ = 59.5; *P* < 0.0001). The same pattern was observed with DEM: DEM pre-exposure 92.1 ± 3.2% vs. 51.9 ± 8.5% for no DEM pre-exposure (*χ*^2^ = 38.6; *P* < 0.0001) (Fig. 1B). Synergist bioassay with DEF also revealed significant recovery of susceptibility to clothianidin (DEF pre-exposure 86.4 ± 7.1% vs. 51.9 ± 8.5% mortality without DEF pre-exposure; *χ*^2^ = 28.6; *P* < 0.0001) although this was slightly lower compared to PBO and DEM. All these results suggest that monooxygenases, GSTs and esterases all combined to drive the reduced susceptibility clothianidin resistance in Nkolondom.

### Diagnostic dosage of clothianidin using acetone and MERO as a solvent

**Fig. 3:**
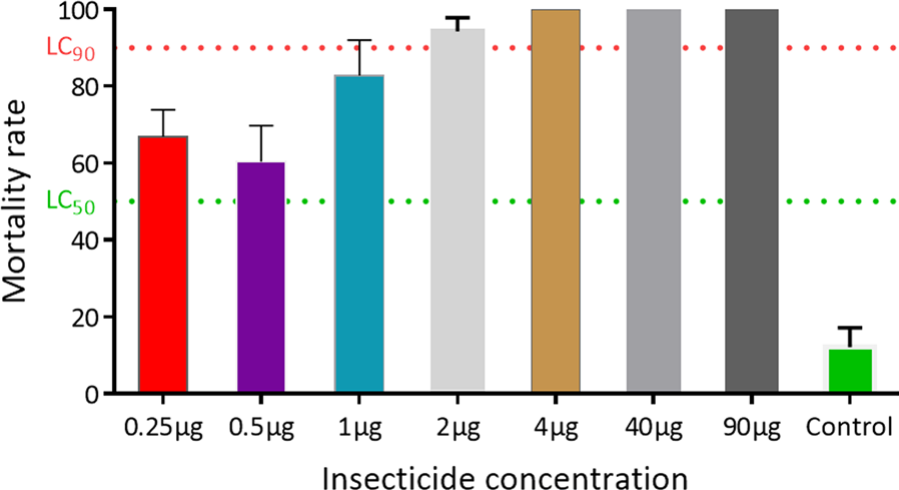
Assessment of diagnostic dose of clothianidin using acetone and MERO as solvent. Percentage mortality (24 h) of the susceptible lab strain Kisumu after exposure to each of the six concentrations of clothianidin (with acetone + MERO as solvent). LC_50_ represents the concentration able to kill 50% of mosquitoes and LC_90_ the concentration able to kill 90%

Because of the very high mortality consistently observed with clothianidin dissolved in acetone + MERO, we decided to establish the diagnostic concentration using the susceptible lab strain Kisumu. The mortality 24 h post-exposure to clothianidin were ranged from 66.8 ± 7.0% at 0.25 µg/ml, 60.9 ± 9.4% at 0.5 µg/ml, 82.8 ± 9.3% at 1 µg/ml to 94.2 ± 3.6% at 2 µg/ml and 100% at 4 µg/ml, 40 µg/ml and 90 µg/ml (Fig. 3). These results suggest that the concentration of 4 µg/ml (as 2 µg/ml kills more than 90% of the susceptible strain) could be used as a diagnostic dose for resistance monitoring in the field populations of malaria vectors.

### Cross-resistance between clothianidin and pyrethroids

**Fig. 4:**
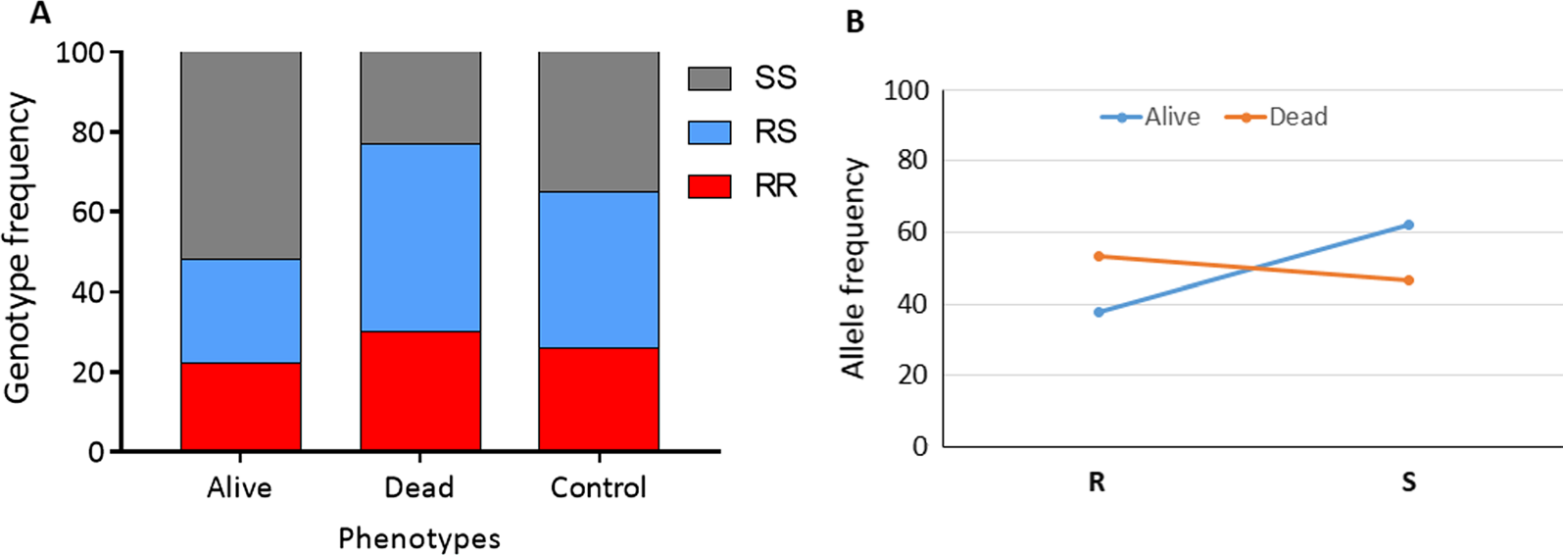
Association between the L1014F-kdr_w mutation and resistance to clothianidin. Distribution of genotypes **A** and alleles **B** among the dead and alive mosquitoes after exposure to clothianidin. R represents the 1014F-resistant allele while S represent the L1014 susceptible allele

**Fig. 5:**
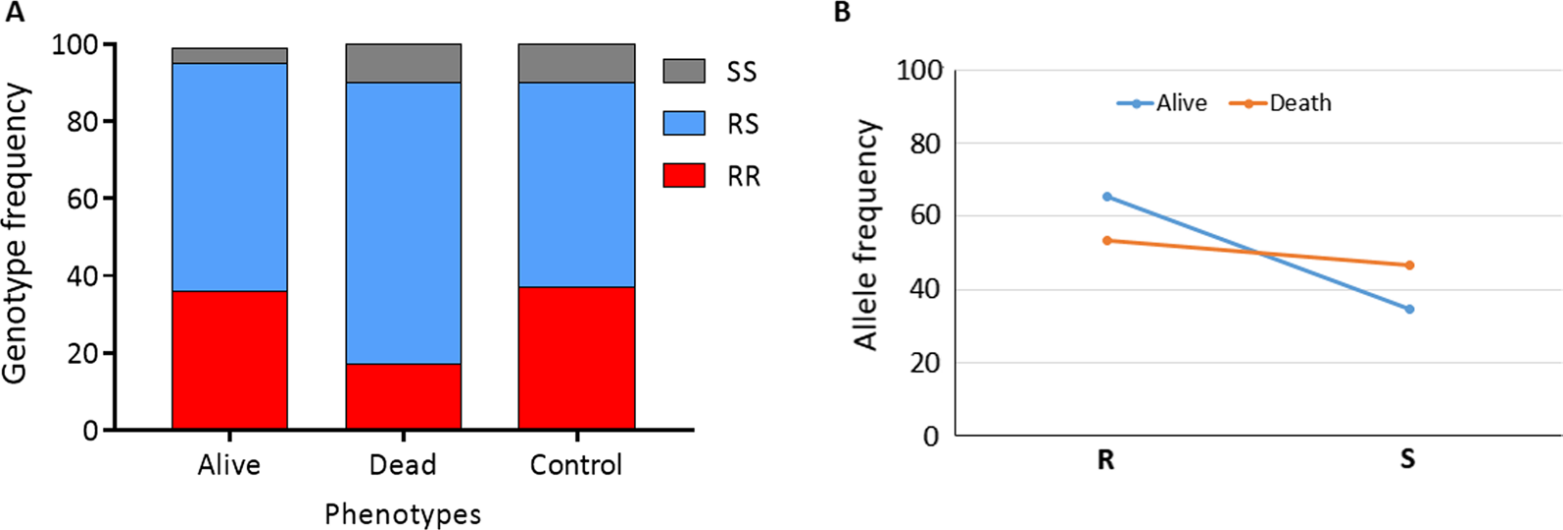
Association between the I114TF-GSTe2 mutation and resistance to clothianidin. Distribution of genotypes **A** and alleles **B** among the dead and alive mosquitoes after exposure to clothianidin. R represents the 114T-resistant allele while S represents the I114 susceptible allele

The distribution of L1014F-Kdr_w genotypes in mosquitoes alive after exposure to a sub-lethal dose of clothianidin was as follows: 24.1% (7/29) homozygous resistant (1014F/F), 27.6% (8/29) heterozygotes (L1014F-RS) and 55.17% (16/29) homozygous susceptible (L/L1014) (Fig. 4). In the dead mosquitoes, 30.0% (9/30) were homozygous resistant (1014F/F), 46.7% (14/30) were heterozygotes (L1014F-RS) whereas 23.3% (7/30) were homozygous susceptible (L/L1014). A significant difference was observed in the distribution of L1014F-Kdr_w genotypes between alive and dead mosquitoes (*χ*^2^ = 18.5; *P* < 0.0001) with the homozygote resistant mosquitoes less able to survive (*OR* = 0.5; 95% *CI*: 0.3–0.9; *P* = 0.02) compared to the susceptible genotypes (Table 1). In contrast, a significant difference was observed in the distribution of the I114T-GSTe2 genotypes between dead and alive mosquitoes and those with resistant allele had more ability to survive clothianidin exposure (*χ*^2^ = 9.78; *P* = 0.007) (Figs. 4 and 5). Assessing of the odds-ratio confirmed that mosquitoes with the 114T resistant allele have a significantly greater ability to survive compared to those with the I114 allele as homozygous resistant mosquitoes were significantly more likely to survive clothianidin exposure compared to both heterozygote (*OR* = 2.10; 95% *CI*: 1.11–3.97; *P* = 0.013) and homozygous susceptible (*OR* = 2.46; 95% *CI*: 1.15–5.26; *P* = 0.012) mosquitoes (Table 2). There was no difference between heterozygote and susceptible mosquitoes (*OR* = 1.17; *P* = 0.41).

**Table 1:** Assessment of the association between L1014F-Kdr genotypes/alleles and the ability of mosquitoes to survive clothianidin exposure

Genotypes	L1014F-Kdr and clothianidin resistance	
	Odds ratio	*P*-value
RR vs SS	0.3 (0.2‒0.7)	0.002**
RR vs RS	1.4 (0.7‒2.8)	0.2
RS vs SS	0.2 (0.1‒0.5)	< 0.0001***
R vs. S	0.5 (0.3‒0.9)	0.02*

*R* resistant, *S* susceptible, *SS* homozygote susceptible, *RR* homozygote resistant, *RS* heterozygote; * *P *< 0.05, **P < 0.01, ***P < 0.0001

**Table 2:** Assessment of the association between I114T-GSTe2 genotypes/alleles and the ability of mosquitoes to survive clothianidin exposure

Genotypes	I114T-GSTe2 and clothianidin resistance	
	Odds ratio	*P*-value
RR vs. SS	4.5 (1.3‒15.4)	0.0006**
RR vs. RS	2.5 (1.2‒4.9)	0.01*
RS vs. SS	1.8 (0.6‒5.6)	0.4
R vs. S	1.6 (0.9–2.9)	0.05*

*R* resistant, *S* susceptible, *SS* homozygote susceptible, *RR* homozygote resistant, *RS* heterozygote; * *P < *0.05, **P < 0.01, ***P < 0.0001

## Discussion

The present study compared the efficacy of neonicotinoids in the major malaria vector *An. gambiae* s.l. across many African countries using three different solvents. Susceptibility testing confirmed that neonicotinoids are slower-acting insecticides (when using acetone or ethanol alone) compared with neurotoxic pyrethroids [17]. While pyrethroids are characterized by rapid mortality of mosquitoes within 24 h, clothianidin/imidacloprid/acetamiprid dissolved in ethanol or acetone alone induced mortality at 24 h post-exposure was generally low (albeit highly variable) and increased over days. Final mortality was recorded seven days post-exposure when using ethanol/acetone alone as done previously [10]. The mortality was low when using ethanol and acetone alone and was the lowest in Nkolondom, an area of intense agriculture. The mortality rates of less than 20% with ethanol and less than 40% with acetone observed in this locality support a recent report of reduced susceptibility to neonicotinoids in the *An. gambiae* population of this location [18]. However, the addition of 0.11% MERO® (81% rapeseed oil methyl ester) increased significantly the effect of these insecticides with 100% mortality for all the populations 24 h post exposure even at a very low concentration. The low knock-down/mortality observed for all the populations when using ethanol or acetone alone as a solvent could be linked to the crystallization issue reported for neonicotinoids [19] preventing complete knockdown/mortality within 1‒2 h. The use of MERO® by preventing the crystallization, keeps the neonicotinoids for a longer period in a soluble state and increases the reliability of the bottle assay.

Despite the low mortality obtained with acetone or ethanol alone, a significant difference was observed between different populations with a mortality rate of 11.4% observed in Nkolondom, 31.1% in Mangoum, 45.6% in Ghana and Uganda compared to 71% in Njombe or 89.3% in Congo and 100% in Kisumu. This indicates that using ethanol or acetone alone might still be useful in capturing the variability between populations and even to detect those populations with reduced susceptibility and thus allow a better management of resistance. In contrast, using acetone plus MERO although more suitable in showing the full efficacy of neonicotinoids, might mask the selection of resistance in populations if not used at the right dose and could prevent detecting the emergence of resistance. Therefore, to take advantage of the strengths of both profiles it could be beneficial to monitor the susceptibility profile to neonicotinoids using acetone (or ethanol) alone and also acetone plus MERO. Because the dose of 90 µg/ml as recommended by Bayer or 50 µg as determined recently in Ivory coast is very high for monitoring of resistance, one option could be to reduce this dose to a level allowing to assess the efficacy of neonicotinoids while monitoring resistance development. In this study, we observed that the concentration of 4 µg/ml could be used for monitoring of resistance to clothianidin as 2 µg was giving more than 90% mortality with the susceptible lab strain Kisumu.

### Cross resistance between pyrethroid and neonicotinoid

A negative association was observed between the 1014F-kdr allele and resistance to clothianidin. Such negative impact could be attributed to the deleterious effect of *kdr* or other related genes in the presence of clothianidin, for which the *vgsc* is not the target. Accordingly, a gradual decrease of *kdr*-resistant homozygotes mosquitoes was observed during the clothianidin selection process by Zoh et al. [20] and this could explain the negative correlation observed in this study between *kdr* and clothianidin resistance. This is the first time such negative association is observed between pyrethroid resistance mechanism and insecticides with different mode of action. However, more studies are needed to clarify the link between *kdr* and clothianidin resistance.

Synergist assay using PBO and DEF also revealed significant recovery of susceptibility to clothianidin showing that monoxygenases, GSTs and esterases are all involved in clothianidin resistance. Over expression of P450s monoxygenases was frequently reported in many resistant cases such as in *An. gambiae* recently where a strong selection signature associated with clothianidin selection was observed on a cytochrome P450 gene cluster with the gene *CYP6M1* showing the highest selection signature together with a transcription profile supporting a role in clothianidin resistance. Overexpression of P450s were also mentioned in *Myzus persicae* [21], *Bemisia tabaci* [22–24], *Trialeurodes vaporariorum* [25], *Nilaparvata lµgens* [26], *Leptinotarsa decemlineata* [27] and many other pests [28].

For the first time, we observed in this study a significant correlation between *GSTe2* and clothianidin resistance in *An. gambiae* indicating that *GSTe2* could metabolise or could be involved in phase 2 conjugation of metabolite of clothianidin degradation. This can be supported by the synergist testing with DEM which helped to recover the susceptibility to clothianidin. Since *GSTe2* is also pyrethroid/DDT resistance gene, its association with clothianidin resistance indicates that clothianidin-based tools could probably show reduced efficacy in areas of high GSTe2-based metabolic resistance. However, future studies are needed to establish the role of GST or specific mechanisms such as esterases and P450s in clothianidin resistance.

One limitation of the study is that we did not evaluate the susceptibility profile of field mosquitoes with 4 µg/ml which was found as a diagnostic concentration (with acetone + Mero) but this will be done in the future.

## Conclusions

This study investigated the susceptibility of the major African malaria vector *An. gambiae* to neonicotinoids using three different solvents and evaluated potential cross resistance between pyrethroid resistance markers and clothianidin survival. The study revealed that the use of acetone or ethanol alone as a solvent for clothianidin can over-estimate the level of resistance in mosquitoes due to crystallisation issue. Furthermore, we showed that the use of clothiandin dissolved in acetone + MERO display very strong efficacy with 4 µg/ml as diagnostic dose for monitoring of resistance to clothianidin but could mask the development of resistance. We recommend therefore monitoring the susceptibility using both acetone alone and acetone + MERO to capture the accurate resistance profile of the mosquito populations.

## Supplementary Information

**Additional file 1.** Variation in mortality rate of the lab strain kisumu over 7days after exposure to clothianidin and imidacloprid with different solvents.

**Additional file 2.** Variation in mortality rate of *An gambiae* populations across Africa over 7days after exposure to clothianidin with different solvents.

**Additional file 3.** Variation in mortality rate of *An gambiae* populations from Ghana and Uganda over 7days after exposure to clothianidin with different solvents.

## Data Availability

All the relevant datasets supporting the conclusions of this article are included within the article.
