# Supplementary figures and images for: Comparative study of the effect of solvents on the efficacy of neonicotinoid insecticides against malaria vector populations across Africa
Source: Infect Dis Poverty. 2022 Apr 25;11:35. doi: 10.1186/s40249-022-00962-4 (PMC9036736; doi:10.1186/s40249-022-00962-4)

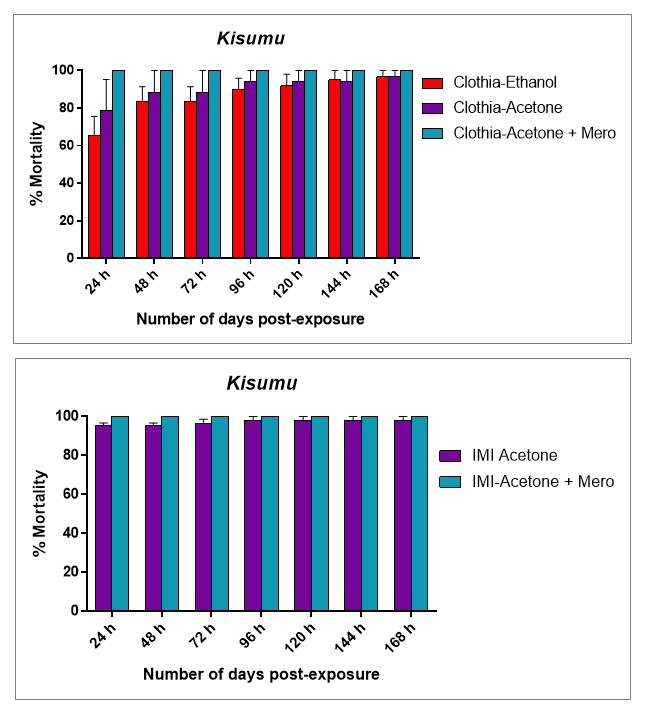

Supplement: Supplementary file 1 — Additional file 1. Variation in mortality rate of the lab strain kisumu over 7days after exposure to clothianidin and imidacloprid with different solvents. [file 40249_2022_962_MOESM1_ESM.tif]

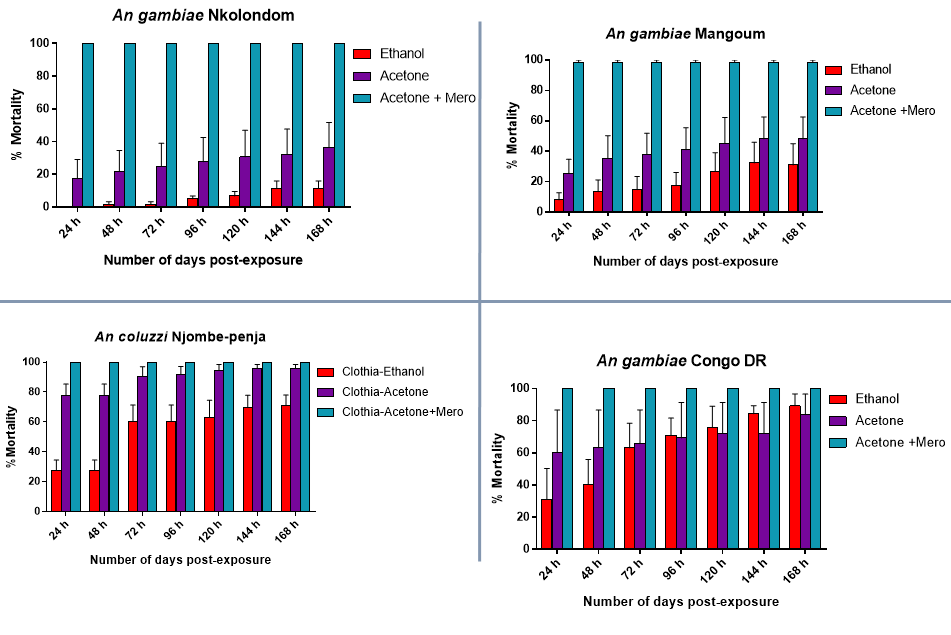

Supplement: Supplementary file 2 — Additional file 2. Variation in mortality rate of An gambiae populations across Africa over 7days after exposure to clothianidin with different solvents. [file 40249_2022_962_MOESM2_ESM.tif]

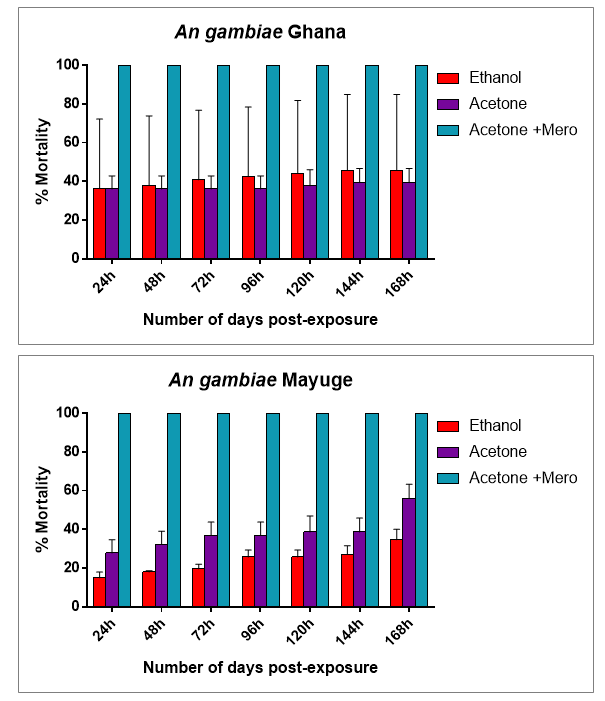

Supplement: Supplementary file 3 — Additional file 3. Variation in mortality rate of An gambiae populations from Ghana and Uganda over 7days after exposure to clothianidin with different solvents. [file 40249_2022_962_MOESM3_ESM.tif]
